# Serious adverse events and 30-day hospital readmission rate following elective total knee arthroplasty: a systematic review and meta-analysis

**DOI:** 10.1186/s13018-021-02358-w

**Published:** 2021-03-31

**Authors:** Costas Papakostidis, Peter V. Giannoudis, J. Tracy Watson, Robert Zura, R. Grant Steen

**Affiliations:** 1 Orthopaedic Department, Limassol General Hospital, Limassol, Cyprus; 2 Academic Department of Trauma & Orthopaedics, School of Medicine, University of Leeds, Leeds, UK; 3 NIHR Leeds Biomedical Research Unit, Chapel Allerton Hospital, Leeds, UK; 4 Department Of Orthopedic Surgery, University of Arizona College of Medicine, Phoenix, AZ USA; 5 Department of Orthopedic Surgery, Louisiana State University Medical Center, New Orleans, LA USA; 6 Present address: 103 Van Doren Place, Chapel Hill, NC 27517 USA

**Keywords:** Return to surgery, Venous thromboembolism, Infection

## Abstract

**Background:**

Elective total knee arthroplasty (TKA) is a common surgery which has evolved rapidly. However, there are no recent large systematic reviews of serious adverse event (SAE) rate and 30-day readmission rate (30-dRR) or an indication of whether surgical methods have improved.

**Methods:**

To obtain a pooled estimate of SAE rate and 30-dRR following TKA, we searched Medline, Web of Science, Cochrane Library, and Google Scholar databases. Data were extracted by two authors following PRISMA guidelines. Eligibility criteria were defined prior to a comprehensive search. Studies were eligible if they were published in 2007 or later, described sequelae of TKA with patient *N* > 1000, and the SAE or 30-dRR rate could be calculated. SAEs included return to operating room, death or coma, venous thromboembolism (VTE), deep infection or sepsis, myocardial infarction, heart failure or cardiac arrest, stroke or cerebrovascular accident, or pneumonia.

**Results:**

Of 248 references reviewed, 28 are included, involving 10,153,503 patients; this includes 9,483,387 patients with primary TKA (pTKA), and 670,116 patients with revision TKA (rTKA). For pTKA, the SAE rate was 5.7% (95% CI 4.4−7.2%, *I*^2^ = 100%), and the 30-dRR was 4.8% (95% CI 4.3−5.4%, *I*^2^ = 100%). For rTKA, the SAE rate was 8.5% (95% CI 8.3−8.7%, *I*^2^ = 77%), while the 30-dRR was 7.2% (95% CI 6.4−8.0%, *I*^2^ = 81%). Odds of 30-dRR following pTKA were about half that of rTKA (OR 0.57, 95% CI 0.53−0.62%, *p* < 0.001, *I*^2^ = 45%). Of patients who received pTKA, the commonest SAEs were VTE (1.22%; 95% CI 0.83−1.70%) and genitourinary complications including renal insufficiency or renal failure (1.22%; 95% CI 0.83−1.67%). There has been significant improvement in SAE rate and 30-dRR since 2010 (χ^2^ test < 0.001).

**Conclusions:**

TKA procedures have a relatively low complication rate, and there has been a significant improvement in SAE rate and 30-dRR over the past decade.

**Supplementary Information:**

The online version contains supplementary material available at 10.1186/s13018-021-02358-w.

## Introduction

Serious adverse events (SAEs) can be an indicator of therapeutic failure or declining patient health and are costly to the medical system [1]. Because SAEs represent an opportunity to improve patient medical care [2], reimbursement policies emphasize reducing the SAE rate associated with specific medical facilities and defined medical services [3]. The Affordable Care Act in the USA mandated reporting of SAEs and established the 30-day readmission rate (30-dRR) as an important metric [4]. Hospital 30-dRR is now widely used as a surrogate measure of healthcare quality by the Centers for Medicare & Medicaid Services [5]. The emphasis on characterizing healthcare quality has resulted in a spate of recent papers that have assessed the SAE rate and the 30-dRR for total knee arthroplasty, although these analyses often include fewer than 10,000 patients [6–8].

There is heterogeneity in the reported complication rate associated with total knee arthroplasty (TKA), which may arise because of the relatively small samples studied. For example, using the American College of Surgeons National Surgical Quality Improvement Program (NSQIP) database for primary total knee arthroplasty (pTKA) for the years from 2011 to 2013, the SAE rate was 1.4% among 65,694 patients [6], while the 30-dRR was 3.5% among 6790 patients [7]. As for revision total knee arthroplasty (rTKA), again using the NSQIP database from 2011 to 2013, the rate of SAEs was 2.7% among 4911 patients [6], while the 30-dRR was 6.4% among 4977 patients [8]. Several factors could impact these metrics, including the era in which TKA was done, procedural complexity, medical comorbidities, and patient age, so small sample sizes can yield unstable estimates.

Current literature lacks a large systematic review of the serious adverse event (SAE) rate and the 30-day readmission rate (30-dRR) for TKA. Our primary aim is to provide a pooled estimate of effect size for both SAE rate and 30-dRR following elective TKA. Secondary aims of this systematic review are to evaluate causes of and risk factors for SAEs and 30-dRR, and to determine whether there have been recent reductions in SAE rate or 30-dRR.

## Methods

This systematic review and meta-analysis adhered to the Preferred Reporting Items for Systematic Reviews and Meta-Analyses (PRISMA) guidelines [9].

### Eligibility criteria and literature search

Eligibility criteria were defined prior to a comprehensive search of the relevant literature and were formulated according to the PICO format. Studies were considered eligible if they met the following inclusion criteria:

- *Participants* – Adult patients derived from large cohort studies including at least 1000 patients and published in 2007 or later
- *Intervention* – Primary total knee arthroplasty (pTKA) (unilateral or bilateral) or revision total knee arthroplasty (rTKA)
- *Comparator* – Comparison, when possible, between various subgroups of an index cohort
- *Outcome* – Serious adverse event (SAE) rate within 30 days following the surgical procedure and 30-day hospital readmission rate (30-dRR). SAEs included the following conditions: return to the operating room (OR), death or coma, venous thromboembolism (VTE), deep infection or sepsis, myocardial infarction (MI), heart failure (HF) or cardiac arrest, stroke or cerebrovascular accident, or pneumonia. A “30-day readmission” was defined as an admission to any service of any hospital within 30 days of a TKA procedure or discharge from an orthopedic service following a TKA procedure.

Exclusion criteria were studies reporting strictly on a subgroup of adverse events (e.g., mortality rate, MI rate following TKA, etc.), studies with inadequate data for both outcomes of interest, studies published prior to 2007, studies with less than 1000 participants, and experimental or biomechanical studies.

An electronic search of the Medline database via the PubMed search engine was initially conducted by two independent researchers (CP, RGS) using the following Medical Subject Headings (MeSH) terms and Boolean operators: (total knee arthroplasty OR total knee replacement) AND (thirty day OR 30 day*) AND (readmission* OR patient readmission) AND (complication* OR adverse event OR outcome*). The search was further extended to the Web of Science, Cochrane Library, and Google Scholar databases. In addition, the reference sections of all eligible articles discovered in the initial electronic search were then manually searched, as was the reference section of a recent meta-analysis [3], to yield articles that had been potentially missed by the initial search. No language restrictions were imposed. Titles of journals, names of authors, and institutions were not masked, to avoid duplication of data. The reviewers independently assessed the titles and abstracts of all retrieved articles and, for potentially eligible articles, the full text was obtained and screened against the eligibility criteria. Any disagreement between the reviewers was resolved by discussion. The search was completed on February 21, 2020 and was limited to the time period since 1 January 2007.

### Data extraction

The following data were extracted from each eligible paper and tabulated into a predefined spreadsheet: demographic data and baseline characteristics, sample size, data source, enrollment period, type of procedure (pTKA, rTKA), number of SAEs, number of hospital readmissions within 30 days, and causes and risk factors for 30-day readmissions or SAEs. The data source was categorized as single hospital database, multicenter registry database (collecting data from more than two hospitals), and nationwide databases (Veteran Affairs [VA], Center for Medicare and Medicaid Services [CMS], National Surgical Quality Improvement Program [NSQUIP]). Patients’ enrollment was categorized into two periods: before 2010 and after 2010. Risk factors for the primary outcomes of interest were recorded from relevant studies when available, along with the respective statistical correlation based on multivariate analysis. Causes for 30-day readmissions and SAEs were collected from relevant studies and pooled appropriately (as described in the statistical section).

### Assessment of the risk of bias

Risk of bias was evaluated across all primary studies using the Quality in Prognosis Studies (QUIPS) tool [10]. The overall risk of bias was ascertained by rating each of the six component domains of the tool, namely (1) study participation, (2) attrition, (3) prognostic factor measurement, (4) outcome measurement, (5) confounding, and (6) statistical analysis and reporting. For every primary study, each domain of the QUIPS tool was given a rating of either low, moderate, or high risk of bias, based on certain prompting items and considerations provided by the tool.

Each risk factor for the main outcomes of interest was assessed in terms of quality of evidence based on the adjusted Grading of Recommendations Assessment, Development and Evaluation (GRADE) framework [11]. The GRADE framework provides 7 factors, each one being rated as either “no serious limitations” or “serious limitations”. A risk factor that had 5 or more scores of “no serious limitations” were considered high quality. Those with 3 or 4 scores of “no serious limitations” were considered “moderate quality”, while risk factors with less than 3 scores of “no serious limitations” were defined as low quality.

### Statistical analysis

All outcomes of interest obtained from studies without a comparator cohort were expressed as proportions (e.g., SAE rate or 30-dRR). Pooling of proportions was done with the MedCalc software (version 14.8.1) using a random effects model, as we assumed that the cohorts within the primary studies were not identical, so the true effect size was not the same across those studies. Statistical heterogeneity was detected with the use of Cochran’s Q test and Higgins I^2^ test [12, 13]. The level of statistical significance was set at 0.1 for the Q test (as it is characterized by low sensitivity for detecting heterogeneity). The I^2^ test is bound at its upper end by 100% and values of 25, 50, and 75% were thought to represent low, moderate, and high degrees of heterogeneity, respectively. Only in the complete absence of statistical heterogeneity (*I*^2^ = 0) would results of the pooling process using the fixed effect model be valid. Comparison between proportions was done with the χ^2^ test.

For studies with comparator cohorts, binary outcomes of interest were expressed as odds ratios (ORs) with respective 95% confidence intervals (95% CIs). Pooling of data was done with the RevMan (5.3) software (Review Manager, Nordic Cochrane Centre, Copenhagen, Denmark) using the Inverse Variance statistical method and a fixed or random effects model, based on our previous assumptions. The results of pooling were expressed graphically as forest plots. In addition, appropriate funnel plots were generated to investigate the potential presence of publication bias. We utilized the Comprehensive Meta-analysis V3 software (Biostat) to generate the respective funnel plot for the 30-dRR. Furthermore, the Duval and Tweedie’s Trim and Fill test was used to impute potentially missing studies, as well as Orwin’s fail-safe N for a quantitative measurement of the publication bias. For binary outcomes based on studies with comparator groups, the RevMan 5.3 software was used to generate inverse funnel plots.

#### Subgroup analysis

In order to explore the effect of the potential presence of heterogeneity on the final outcomes, certain subgroups of the initial cohort were determined, and the outcomes of interest were calculated within each subgroup. These subgroups were revision knee arthroplasties, unilateral knee arthroplasties, and bilateral knee arthroplasties.

### Sensitivity analysis

Sensitivity analysis was performed by repeating the pooling process after eliminating studies of very large size, that would potentially affect the final outcomes by being overly weighted, and studies with a low rating by the QUIPS tool.

## Results

**Fig. 1:**
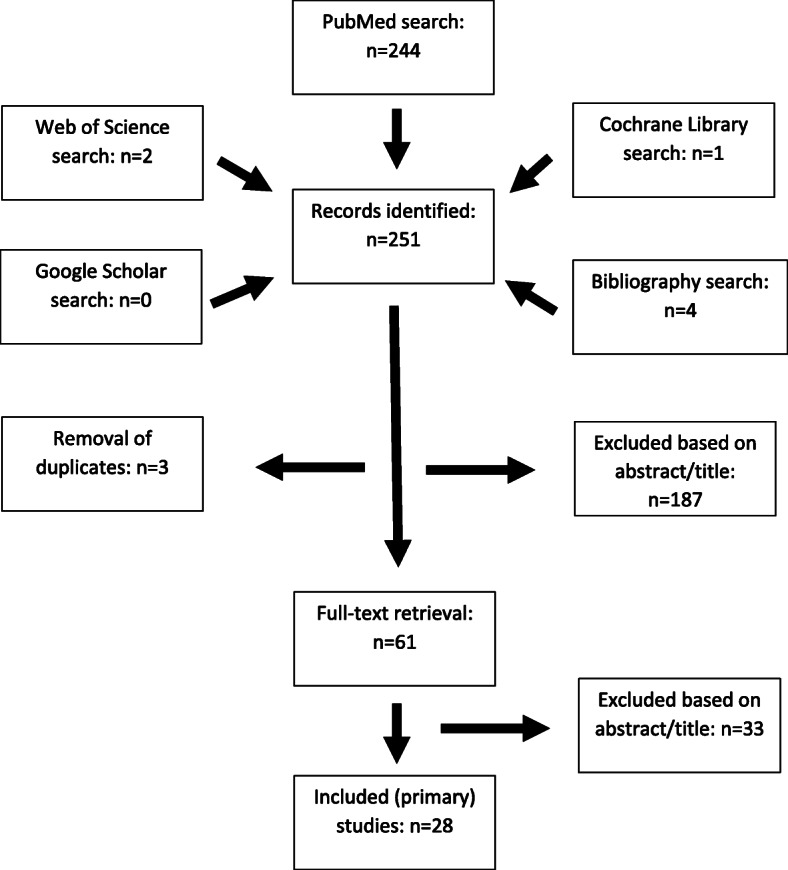
PRISMA flow chart of literature review

The initial PubMed search yielded 244 citations. Three additional records were identified through the electronic search of other electronic databases, while 4 records were found through a manual search of relevant bibliographies. After duplicates were removed, 248 abstracts and abstract titles were screened for suitability. Most studies (187) were excluded based on information provided in the title and abstract. Sixty-one articles were ultimately retrieved for full-text review. After applying eligibility criteria, 33 articles were excluded, leaving 28 primary studies [6–8, 14–38] for analysis (Table 1), as summarized in a PRISMA flow chart (Fig. 1, see also PRISMA checklist, additional file).

**Table 1:** Evidentiary table

Author [refs]		Publication	Procedure	Data source	Enroll-ment period	No. of patients	SAEs	30-day RR
1	**Ross TD** **[**14**]**	J Arthro, 2020	Primary TKR	Regional database, Ontario, Can (IC/ES)	2003−2016	200,421	NR	6819
			Rev TKA			4731	NR	289
2	**Lehtonen EJ** **[**15**]**	Acta Orthop Bras 2018	Primary TKR	ACS-NSQIP	2012−2015	137,209	6143	4668
3	**Ali AM** **[**16**]**	J Arthro 2018	Primary TKR	HES	2006−2015	566,323	NR	35,252
			Unicomp			40,650	NR	1424
			PF			11,442	NR	519
4	**Bottle A** **[**17**]**	J Arthro 2018	Primary TKR	HES	Apr 2010−March 2015	311,033	NR	18,814
5	**D'Apuzzo** **[**18**]**	JBJS Am 2017	Primary TKR	SPARCS	1997−2014	377,705	NR	22,076
6	**Yao DH** **[**19**]**	J Arthro. 2017	Primary TKA	NSQIP	2011−2014	71,293	2490	1952
7	**Keswani A** **[**8**]**	J Arthro. 2016	Rev TKA	NSQIP	2011−2013	4977	397	NR
8	**Belmont PJ** **[**20**]**	Knee Surg Sports Traumatol Arthrosc. 2016	Rev TKA	NSQIP	2011−2012	1754	NR	108
9	**Hart A** **[**7**]**		Bilat TKA	NSQIP	2011−2013	1771	67	64
		J Arthro 2016	Unilat TKA			6790	151	240
10	**Bohl DD** **[**6**]**	J Arthro. 2016	Primary TKA	NSQIP	2011−2013	65,694	920	2956
			Rev TKA			4911	133	363
11	**Culler SD** **[**21**]**	J Arthro, 2015	Primary TKA	MedPAR	2011	353,650	41,792	NR
12	**Raines BT** **[**22**]**	J Arthro 2015	Primary TKA	VA	2005−2009	16,808	1848	1106
13	**Schairer WW** **[**23**]**	CORR 2014	Primary TKA	Single-institution database	2005−2011	1032	NR	64
			Rev TKA			262	NR	34
14	**Bosco JA** **[**24**]**	J Arthro 2014	Primary TKA	Single-hospital database	2009−2012	1263	NR	55
			Rev TKA			118	NR	14
15	**Belmont PJ Jr** **[**25**]**	JBJS Am, 2014	Primary TKA	NSQIP	2006−2010	15,321	851	NR
16	**Pugely AJ** **[**26**]**	J Arthro, 2013	Primary TKA	NSQIP	2011	11,814	NR	543
17	**Zmistowski B** **[**27**]**	JBJS Am 2013	Primary TKA	Institutional Arthroplasty database	2004−2008	5207	NR	199
18	**Pugely AJ** **[**28**]**	JBJS Am 2013	Primary TKA	NSQIP	2005−2010	14,052	1636	NR
19	**Cram P** **[**29**]**	JAMA. 2012	Primary vs Rev TKA	CMS	1991−2010	3,271,851	135,195	145,504
						318,563	27,569	23,074
20	**Cram P** **[**30**]**	Mayo Clin Proc. 2012	Primary TKA	CMS	2006	64,712	5357	5320
21	**Vorhies JS** **[**31**]**	CORR 2012	Primary TKA	Medicare	2002−2007	4057	NR	228
22	**Brown NM** **[**32**]**	J Arthro. 2012	Unicomp	3-Institution database (Multicenter)	2004−2009	605	26	16
			Primary TKA			2235	246	94
23	**Singh JA** **[**33**]**	Arthritis Rheum. 2011	Primary TKA	Regional database, Pennsylvania (PHC4)	2002	19,418	364	NR
24	**Husni ME** **[**34**]**	BMC Musculoskel Disord. 2010	Primary TKA	CMS	2000	9157	390	NR
25	**Huddleston JI** **[**35**]**	J Arthro, 2009	Primary TKA	Medicare	2002−2004	2033	132	NR
26	**Pulido L** **[**36**]**	J Arthro, 2008	Primary TKA	Single-institution database	Jan 2000−Aug 2006	5173	303	NR
			Rev TKA			645	61	NR
27	**Memtsoudis S** **[**37**]**	CORR, 2008	Unilat TKA	NHDS (nationwide database)	1990−2004	3,672,247	299,526	NR
			Bilat TKA			153,259	18,696	NR
			Rev TKA			334,155	29,007	NR
28	**Katz JN** **[**38**]**	Med Care. 2008	Primary TKA	Medicare	2000	80,604	2826	NR

*ACS-NSQIP*, American College of Surgeons National Surgical Quality Improvement Program; *CMS*, Centers for Medicare & Medicaid Services; *HES*, Hospital Episode Statistics; *IC*/*ES*, Institute for Clinical and Evaluative Sciences; *MedPAR*, Medicare Provider Analysis and Review; *NHDS*, National Hospital Discharge Survey; *NR*, not reported; *PF*, patellofemoral; *SPARCS*, Statewide Planning and Research Cooperative System; *VA*, Veteran’s Administration

The total number of included patients across all primary studies was 10,153,503. Of this total, 9,483,387 patients had undergone primary TKA (pTKA), while the remaining 670,116 patients had undergone revision TKA (rTKA). Four studies used data from a single hospital database [23, 24, 27, 36], 1 study was a multicenter registry database [32], and the remaining 23 studies used nationwide databases [14–22, 24–26, 28–31, 33–38] (Table 1). Eighteen studies did not include any comparator group, reporting solely on either pTKAs (16 studies) [15, 17–19, 21, 22, 25–28, 30, 31, 33–35, 38] or rTKAs (2 studies) [8, 20]. The remaining 10 studies included various comparator cohorts (pTKAs vs. rTKAs in 7 studies [6, 14, 23, 24, 29, 36, 37], unilateral versus simultaneously bilateral TKAs in 2 studies [7, 37], and unicompartmental versus pTKAs in 2 studies [16, 32] (Table 1).

### Assessment of the risk of bias

Using the QUIPS tool, all studies were rated as having “low risk of bias” in the study participation domain, while in the remaining domains, studies were assigned scores of low, moderate, or high risk of bias (Table 2).

**Table 2:** The QUIPS tool

Author		Risk in the various QUIPS domains					
		Study participation	Attrition	Prognostic factor measurement	Outcome measurement	Study confounding	Statistical analysis & reporting
1	**Ross TD** **[**14**]**	Low	Low	Low	Low	Low	Low
2	**Lehtonen EJ** **[**15**]**	Low	Low	Low	Moderate	Low	Moderate
3	**Ali AM** **[**16**]**	Low	Low	Low	Low	Low	Low
4	**Bottle A** **[**17**]**	Low	Low	Moderate	Moderate	Moderate	Low
5	**D'Apuzzo** **[**18**]**	Low	Low	Low	Low	Low	Low
6	**Yao DH** **[**19**]**	Low	Low	Low	Low	Low	Low
7	**Keswani A** **[**8**]**	Low	Low	Low	Low	Low	Low
8	**Belmont PJ** **[**20**]**	Low	Low	Low	Low	Low	Low
9	**Hart A** **[**7**]**	Low	Low	Low	Low	Moderate	Low
10	**Bohl DD** **[**6**]**	Low	Low	**High**	Low	Low	Low
11	**Culler SD** **[**21**]**	Low	Moderate	**High**	**High**	**High**	Moderate
12	**Raines BT** **[**22**]**	Low	Low	Low	Low	Low	Low
13	**Schairer WW** **[**23**]**	Low	**High**	Low	Low	Low	Low
14	**Bosco J** **[**24**]**	Low	Low	**High**	**High**	**High**	**High**
15	**Belmont PJ Jr** **[**25**]**	Low	Low	Low	Low	Low	Low
16	**Pugely AJ** **[**26**]**	Low	Low	Low	Low	Low	Low
17	**Zmistowski B** **[**27**]**	Low	Low	Low	Low	Low	Low
18	**Pugely AJ** **[**28**]**	Low	Low	Low	Low	Low	Low
19	**Cram P** **[**29**]**	Low	Low	Moderate	Low	Low	Low
20	**Cram P** **[**30**]**	Low	Moderate	**High**	Low	Low	Low
21	**Vorhies JS** **[**31**]**	Low	Low	**High**	Low	Moderate	Low
22	**Brown NM** **[**32**]**	Low	Low	**High**	**High**	Moderate	Low
23	**Singh JA** **[**33**]**	Low	Low	Moderate	Moderate	Moderate	Moderate
24	**Husni ME** **[**34**]**	Low	Moderate	**High**	**High**	Low	Low
25	**Huddleston JI** **[**35**]**	Low	Low	Moderate	Low	Moderate	Low
26	**Pulido L** **[**36**]**	Low	Low	Moderate	Moderate	Moderate	Moderate
27	**Memtsoudis S** **[**37**]**	Low	Low	Moderate	Low	Moderate	Moderate
28	**Katz JN** **[**38**]**	Low	Low	Moderate	Moderate	Moderate	Low

### Publication bias

**Fig. 2:**
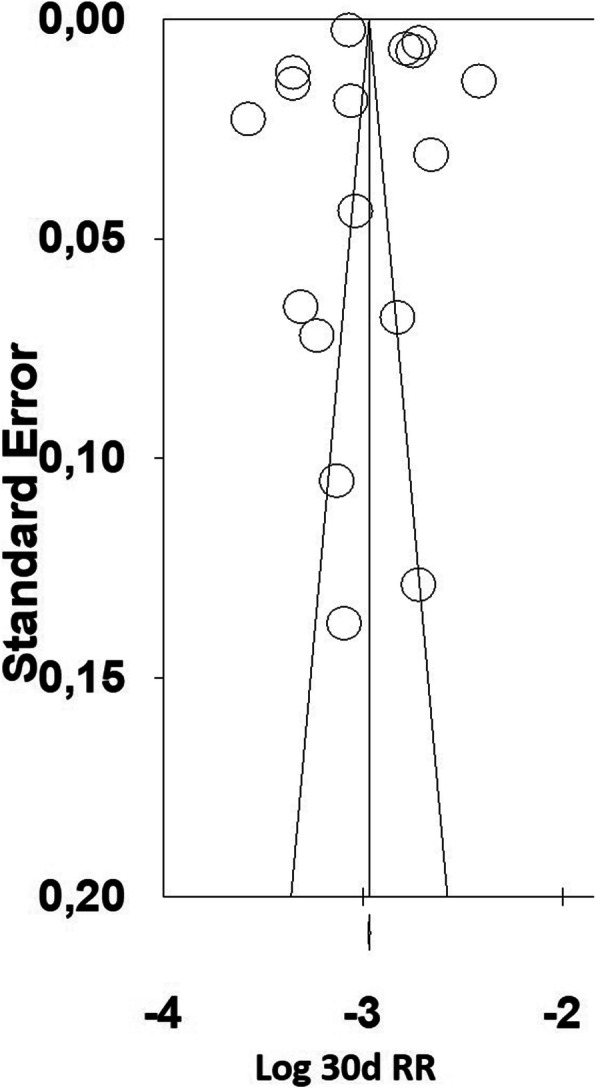
Funnel plot for the 30-day readmission rate data

**Fig. 3:**
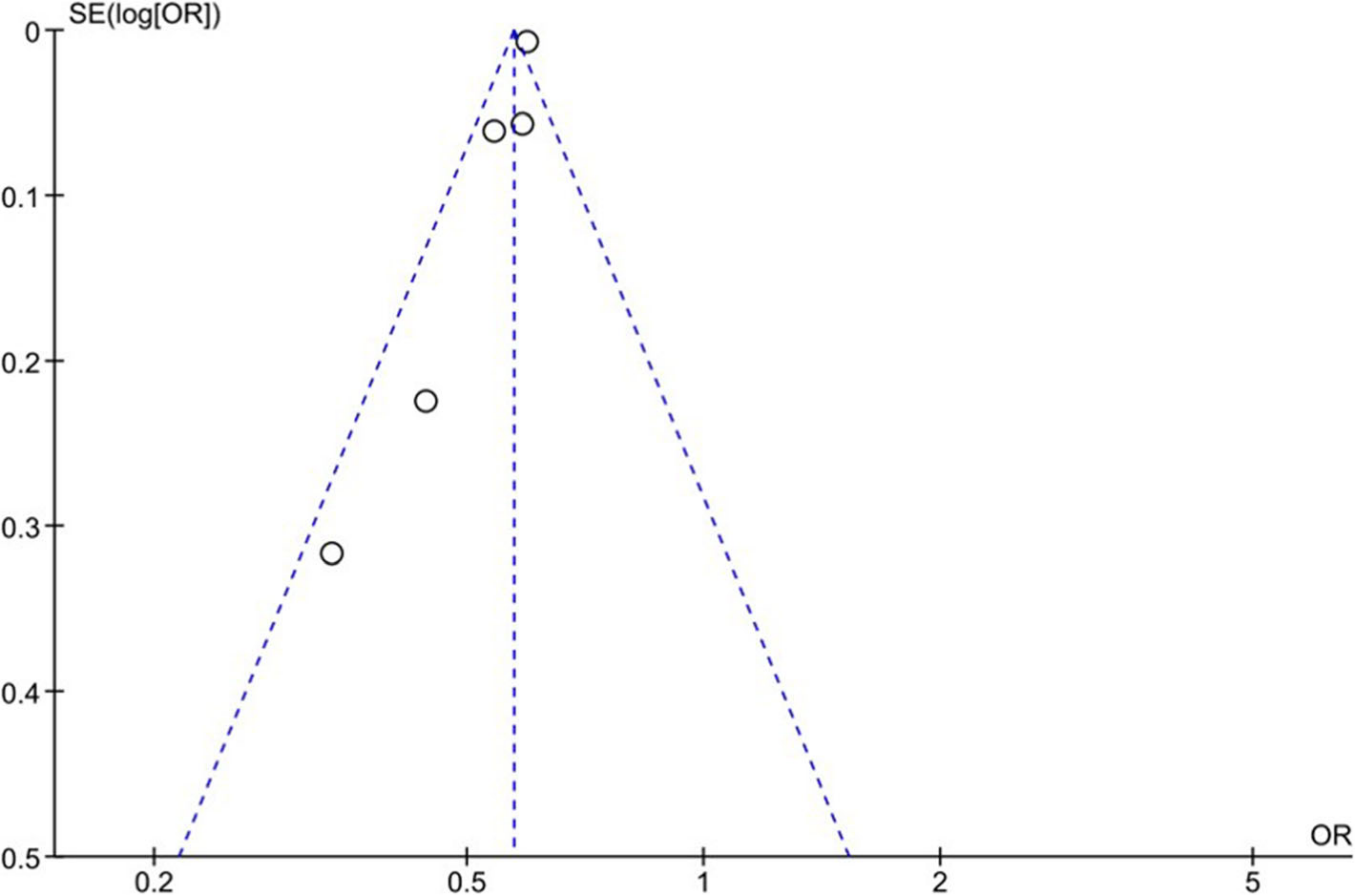
Funnel plot for 30-day readmission rate data of primary TKA vs revision TKA.

We utilized the CMA V3 (Biostat) software to produce a funnel plot for the 30-dRR data. The distribution of data points was almost symmetrical around the calculated weighted mean estimate of effect size, with most primary studies having a large sample size (Fig. 2). A trim and fill test for the random effects model showed that there was no study missing either to the left or to the right of the average, suggesting that missed studies likely would not change our results substantially. Furthermore, Orwin’s fail-safe N indicated that 12 studies with a 30-dRR of 3% would be needed to lower the calculated 30-dRR of 4.8% below 4%. The RevMan 5.3 software funnel plot for 30-dRR of pTKA vs rTKA showed that the distribution of data points was within the confines of the inverse funnel plot but with a tendency for the data points to be to the left of the average line (Fig. 3). This tendency is indicative of literature lacking studies with higher pooled estimate of effect size (odds ratio for readmission rate between primary and revision TKAs).

### Main outcomes of interest

#### Thirty-day readmission rate (30-dRR) for pTKA

**Fig. 4:**
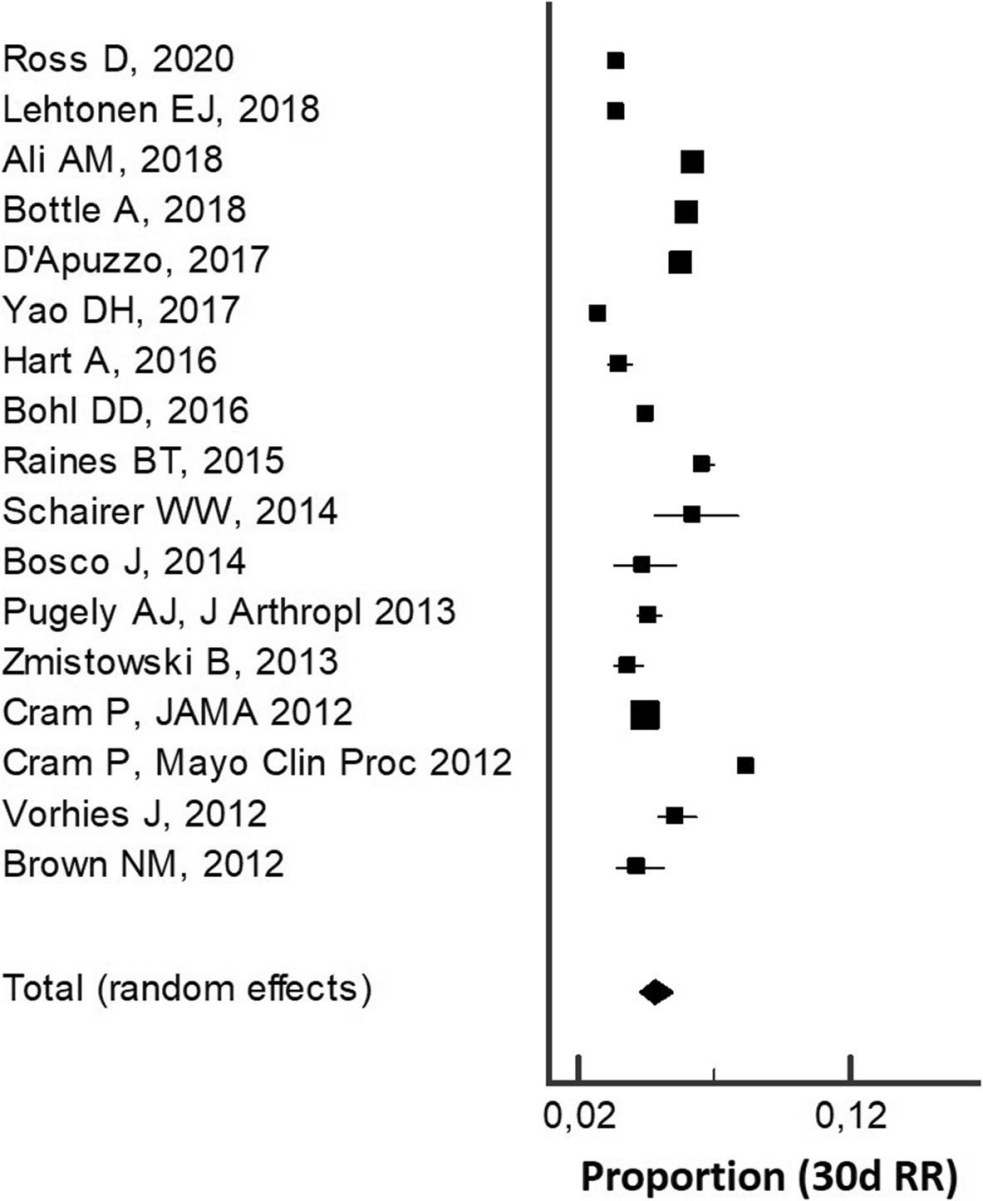
The pooled estimate of effect size for 30-day readmission rate

Relevant data were derived from 17 studies reporting on 5,115,447 patients [6, 7, 14–19, 22–24, 26–31]. The pooled estimate of effect size for 30-dRR, was 4.8% (95% CI 4.3−5.4%, with significant heterogeneity, *I*^2^ = 100%) (Fig. 4).

### Serious adverse event (SAE) rate for pTKA

Data were obtained from 17 studies reporting on 7,808,247 patients [6, 7, 15, 19, 21, 22, 25, 28–30, 32–38]. The pooled estimate of effect size for SAE rate was 5.7% (95% CI 4.4−7.2%, *I*^2^ = 100%). Compared with the 30-dRR, SAE is significantly higher (*p* < 0.001, χ^2^ test).

### Main outcomes of interest in revision total knee arthroplasty (rTKA)

**Fig. 5:**
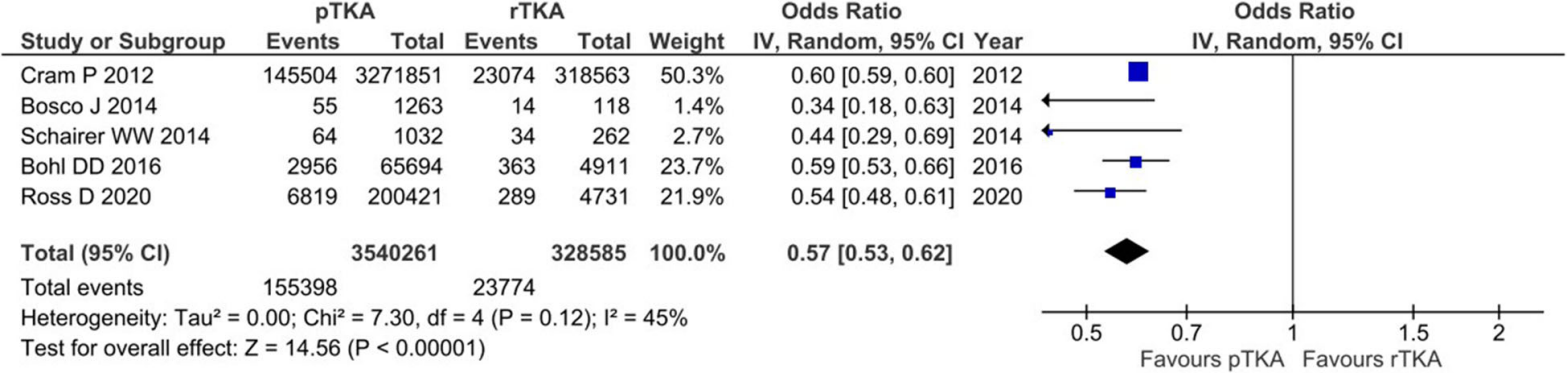
The odd ratios for readmission within 30 days following primary TKA vs revision TKA

**Fig. 6:**
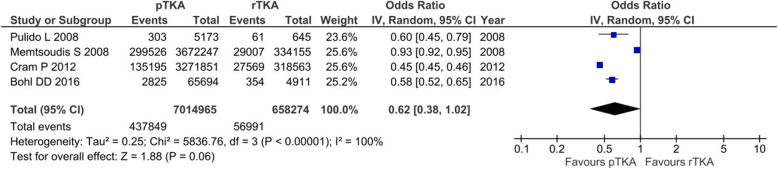
The odds ratios for serious adverse events following primary TKA vs revision TKA

Nine studies, reporting on 670,116 patients, provided relevant data for either 30-dRR or SAE rate in rTKA [6, 8, 14, 20, 23, 24, 29, 36, 37]; 30-dRR was calculated from 6 studies reporting on 330,339 patients [6, 14, 20, 23, 24, 29]. The respective pooled estimate of effect size for 30d-RR was 7.2% (95% CI 6.4−8.0%, *I*^2^ = 81%). The SAE rate was calculated from 5 studies, reporting on 663,251 patients [6, 8, 29, 36, 37]. The pooled estimate of effect size for SAE rate was 8.5% (95% CI 8.3−8.7%, *I*^2^ = 77%). Furthermore, direct comparison between pTKA and rTKA, in terms of 30-dRR and SAE rate, was feasible. With respect to 30-dRR, relevant data were obtained from 5 studies, reporting on 3,540,261 pTKAs and 328,585 rTKAs [6, 14, 23, 24, 29]. The odds of readmission within 30 days following pTKA was about half that of rTKA (OR 0.57, 95% CI 0.53−0.62%, *p* < 0.001, *I*^2^ = 45%) (Fig. 5). Data on SAE rate were abstracted from 5 studies directly comparing 7,014,965 pTKAs and 658,274 rTKAs. Although the difference between pTKA and rTKA favored pTKA, it failed to reach statistical significance (OR = 0.62, 95% CI 0.38−1.02, *p* = 0.06, *I*^2^ = 100%) (Fig. 6).

### Subgroup analysis

#### Bilateral vs unilateral pTKAs

**Fig. 7:**
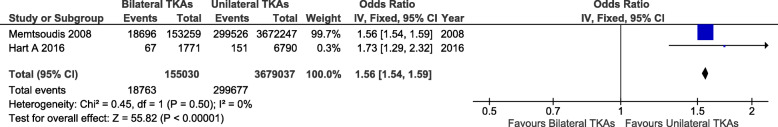
The odds ratios for serious adverse events following unilateral TKA vs bilateral TKA

Two studies comparing 155,030 bilateral TKAs with 3,679,037 unilateral TKAs provided relevant data only for SAE rate [7, 37]. The pooled estimate of effect size favored unilateral TKAs (OR = 1.56, 95% CI 1.54−1.59, *p* < 0.001, *I*^2^ = 0%, fixed effects model) (Fig. 7). Only a single study compared bilateral vs unilateral TKAs in terms of 30-dRR rate and failed to document any statistically significant difference (OR = 1.02, 95% CI 0.8−1.4, *p* = 0.9) [7].

#### Unicompartmental vs pTKAs

Relevant data on 30-dRR were extracted from two studies directly comparing 41,255 unicompartmental TKAs with 568,558 pTKAs [16, 32]. The respective pooled estimate of effect size was in favor of unicompartmental TKA (OR = 0.55, 95% CI 0.52−0.58, *p* < 0.001, *I*^2^ = 0%, fixed effects model). The single study comparing unicompartmental vs pTKA in terms of SAE rate favored the unicompartmental TKA (OR = 0.36, 95% CI 0.24−0.55, *p* < 0.001) [32].

### Time of patient enrollment

The primary outcomes of interest were compared based on when surgery occurred (before 2010 versus 2010 to present) and rates of SAE and 30-dRR were compared (Table 3). A statistically significant reduction in both 30-dRR and SAE rate was documented after 2010 (χ^2^ test; *p* < 0.001) (Table 3).

**Table 3:** Primary outcomes of interest based on time of patients’ enrollment

	Number of studies (Refs)	Sample size	Pooled estimate of effect size (%)	95% CI	Heterogeneity (%)	χ^2^ test
AE rate, before 2010	12	7,173,611	6.5	4.6−8.2	100	*p* < 0.001
AE rate, from 2010 on	6	639,613	4.7	1.7–9.0	100	
30-dRR, before 2010	6	3,364,870	5.4	3.8−7.3	100	*p* < 0.001
30-dRR, from 2010 on	7	605,587	4.3	3.2−5.6	100	

### Causes of SAEs and 30-day readmissions

Causes for both 30-dRR (Table 4) and SAEs (Table 5) following pTKA were explored across primary studies and summarized by pooled analysis. Only relevant causes included in at least 3 primary studies were considered for quantitative synthesis. The most frequent cause of 30-dRRs was VTE (pooled estimate of effect size for VTE: 0.41%; 95% CI 0.26−0.6%), followed by genitourinary (GU) and respiratory complications (Table 4). As for local complications, infections (both deep-periprosthetic and superficial) were the commonest local causes for 30-day readmissions (Table 4). The most frequent causes of adverse events were VTE (pooled estimate of effect size: 1.22%; 95% CI 0.83−1.70%, *I*^2^ = 99.6%) and GU complications (pooled estimate: 1.22%; 95% CI 0.83−1.65%, *I*^2^ = 99.5%), followed by respiratory and cardiac complications (Table 5). As for local causes of SAEs, the commonest was return to OR, followed by local septic complications (Table 5).

**Table 4:** Pooled causes of 30-day readmissions for pTKA

	Number of reporting studies [refs]	Sample size	*N* of events (30d readmissions)	Rate (%)	95% CI	*I*^2^ (%)
**Systemic conditions**						
**VTE**	4 [15, 19, 26, 27]	225,523	881	**0.41**	0.26−0.60	96.4
**GU complications**	3 [15, 19, 26]	220,316	341	**0.18**	0.06−0.34	98.0
**Respiratory complications**	3 [15, 19, 26]	220,316	373	**0.17**	0.09−0.26	94.5
**Cardiac complications**	4 [15, 19, 26, 27]	225,523	239	**0.15**	0.08−0.24	93.2
**Sepsis**	3 [15, 19, 26]	220,316	313	**0.15**	0.05–0.3	98.0
**Stroke / CVA**	3 [15, 19, 26]	220,316	45	**0.02**	0.004−0.05	90.0
**Local conditions**						
**Superficial infection**	3 [15, 19, 26]	220,316	644	**0.32**	0.10−0.65	99.0
**Deep + periprosthetic infection**	3 [15, 26, 27]	154,230	455	**0.56**	0.16−1.21	98.3

*VTE*, venous thromboembolism; *GU*, genitourinary complications (renal insufficiency, renal failure, urinary tract infections [UTI]; *CVA*, cerebrovascular accident. *Respiratory complications*: pneumonia, unplanned intubation, ventilation > 48 h. *Cardiac complications*: myocardial infarction, cardiac arrest

**Table 5:** Pooled causes of SAEs for pTKAs

	Number of reporting studies [refs]	Sample size	*N* of events (SAEs)	Rate (%)	95% CI	*I*^2^ (%)
**Systemic complications**						
**VTE**	11 [7, 15, 19, 21, 25, 28, 30, 33, 35, 36, 38]	770,255	7797	**1.22**	0.83–1.70	99.6
**GU complications**	7 [15, 19, 25, 28, 35–37]	3,917,328	37,180	**1.22**	0.83–1.67	99.5
**Respiratory complications**	10 [7, 15, 19, 21, 25, 28, 35–38]	4,358,372	44,485	**0.45**	0.23–0.76	99.8
**Cardiac complications**	12 [7, 15, 19, 21, 25, 28, 30, 33, 35–38]	4,442,502	42,278	**0.31**	0.12–0.6	99.8
**Sepsis**	7 [6, 15, 19, 25, 28, 30, 36]	373,454	887	**0.26**	0.17–0.38	97.2
**Stroke/CVA**	6 [7, 19, 25, 28, 36, 37]	3,784,876	5433	**0.10**	0.04–0.19	97.2
**Mortality**	12 [6, 7, 19, 21, 25, 28, 30, 33, 35–38]	4,370,987	11,424	**0.16**	0.09–0.24	99.0
**Local complications**						
**Return to OR**	3 [7, 19, 28]	92,135	779	**1.04**	0.57–1.65	97.4
**Superficial infection**	5 [15, 19, 25, 28, 36]	243,048	1066	**0.43**	0.21–0.72	98.6
**Deep + periprosthetic infection**	12 [6, 7, 15, 19, 21, 25, 28, 30, 33, 36–38]	4,506,163	10,591	**0.30**	0.24–0.37	98.3
**Wound dehiscence**	5 [19, 25, 28, 35, 37]	3,774,946	1458	**0.13**	0.05–0.26	98.0
**Implant-related compl.**	4 [19, 28, 35, 36]	92,551	32	**0.04**	0.01–0.08	72.0
**Periprosthetic fractures**	3 [19, 35, 36]	78,499	23	**0.10**	0.008–0.3	91.0

*VTE*, venous thromboembolism; *GU*, genitourinary complications (renal insufficiency, renal failure, urinary tract infections [UTI]; *CVA*, cerebrovascular accident; *OR*, operating theatre. *Respiratory complications:* pneumonia, unplanned intubation, ventilation > 48 h. *Cardiac complications:* myocardial infarction, cardiac arrest.

### Risk factors for either SAE or 30-dRR

Twenty risk factors for SAEs or 30d-RR were identified across primary studies based on multivariate analysis. The most frequently documented risk factors with a positive correlation to the outcome of interest in the primary studies were increased age (13 studies) [14–16, 18, 19, 22, 25–29, 36, 37], male gender (10 studies) [7, 8, 14–16, 18, 19, 26, 29, 37], ASA scores 3−4 (8 studies) [8, 15, 19, 20, 22, 25, 26, 28], and pulmonary disease (8 studies) [7, 8, 18, 19, 22, 26, 28, 35]. Based on the GRADE analysis, 2 of the most frequently stated risk factors (age, ASA 3−4) had minimal bias, while another two (male gender, pulmonary disease) were obtained from studies of moderate quality. Lastly, 12 risk factors were obtained from studies of low quality (Table 6).

**Table 6:** Risk factors for readmission or AEs with GRADE analysis

				GRADE analysis							
Risk factor	Studies with statistically positive correlation (refs)	Studies with no correlation (refs)	Studies not reporting	Study limitations	Inconsistency	Indirect-ness	Impreci-sion	Publica-tion bias	Mod/Large effect size	Dose effect	Overall quality
**Age**	13	2	3	v	v	v	v	v	x	v	(+++)
**Obesity**	6	4	7	v	x	x	x	v	x	x	(+)
**DM**	5	3	11	v	x	x	x	**?**	x	x	(+)
**Cardiac disease**	5	2	11	v	v	x	x	x	x	x	(+)
**Hypertension**	2	4	12	v	x	x	x	x	x	x	(+)
**Previous CVA**	1	2	16	v	x	x	x	x	x	x	(+)
**ASA: 3 - 4**	8	1	9	v	v	v	v	**?**	x	v	(+++)
**Operative time > 135 min**	3	2	14	v	x	x	x	x	x	v	(+)
**LOS > 4 d**	5	0	14	v	v	x	v	x	x	x	(++)
**Female gender**	4	2	2	x	x	v	v	v	x	x	(++)
**Male gender**	10	2	1	v	x	v	v	v	x	x	(++)
**Anesthesia (general vs spinal, epidural, regional)**	2	0	17	v	**?**	x	v	x	x	x	(+)
**Black race**	5	3	11	v	**?**	x	v	x	x	x	(+)
**In-hospital complications**	3	0	15	v	v	x	v	x	v	x	(++)
**Discharge disposition (any facility vs home)**	5	1	13	v	v	x	v	x	x	x	(++)
**Pulmonary disease**	8	1	9	v	v	v	v	**?**	x	x	(++)
**Fluid electrolyte disorder**	2	2	15	x	x	x	x	x	x	x	(+)
**Renal failure**	4	1	14	v	**?**	x	v	x	x	x	(+)
**Simultaneous bilateral TKAs**	2	3	14	x	x	x	x	x	x	x	(+)
**Revision TKA**	4	0	14	x	v	x	v	x	x	x	(+)

GRADE factors: ✓, no serious limitations; ✖, serious limitations (or not present for moderate/large effect size, dose effect); ?, unclear whether limitations are serious; overall quality of evidence: +, low; ++, moderate; +++, high.

The risk factors that had ≥ 5 scores of “no serious limitations” were determined to be high quality. Those that had 3–4 scores of “no serious limitations” were determined to be of moderate quality. Risk factors with < 3 scores of “no serious limitations” were determined to be low quality.

### Sensitivity analysis

To analyze sensitivity, the pooled analysis was repeated after excluding studies with a sample *N* of over 100,000 participants (Table 7). New results did not differ substantially from the original results. The pooling analysis was repeated after excluding studies with at least 2 domains of “high risk” in the QUIPS instrument, and this also did not produce materially different results compared to the original. These findings suggest that our results are robust.

**Table 7:** Results of sensitivity analysis

Outcome of interest	*n* reporting studies	*n* events	OR or rate (%)	95% CI	Statistical model	*I*^2^
30-dRR	11	250,905	4.8	3.5–6.3	RE	99.5%
AEs rate	13	373,290	5.4	3.7–7.4	RE	99.8%
Subgroup analysis						
pTKA vs rTKA: OR, AEs	2	pTKA: 70,867 rTKA: 5556	OR: 0.6	0.5–0.7	FE	0%
pTKA vs rTKA: OR, 30-dRR	4	pTKA: 268,410 rTKA: 10,022	OR: 0.6	0.5–0.6	RE	37%
30-dRR (rTKA)	5	11,776	7.6	6.2–9.0	RE	83%
AEs (rTKA)	3	10,533	8.0	7.0–9.0	RE	60%

*pTKA*, primary TKA; *rTKA*, revision TKA; *RE*, random effects model; *FE*, fixed effects model

## Discussion

Our results show that elective TKA is associated with a significant rate of serious adverse events and hospital readmissions after surgery. The SAE rate for pTKA is 5.7% (95% CI 4.4−7.2%), while the 30d-RR rate is 4.8% (95% CI 4.3−5.4%). For rTKA, the SAE rate is 8.5% (95% CI 8.3−8.7%), while the 30d-RR rate is 7.2% (95% CI 6.4−8.0%). The 30-dRR is significantly lower than the SAE rate for primary and revision TKAs. Primary TKA is associated with fewer hospital readmissions than is revision TKA (Fig. 5), consistent with literature showing that patients with revision TKA, whether for infection or for other causes, are more likely to have an unplanned readmission to the hospital than are patients with primary TKA [23]. The odds of hospital readmission for rTKA were twice as high as those for pTKA, while the odds of SAEs in bilateral TKAs were 1.5 times greater than in unilateral TKAs. Contrasting studies done prior to 2010 with later studies demonstrates a significant reduction in both SAE rate and 30-dRR since 2010 (χ^2^ test; *p* < 0.001) (Table 3).

Hospital readmission after surgery is associated with poor patient outcomes and increased medical costs [39]. Although hospital readmission rates are not highly correlated with mortality rates, short-term readmissions have been identified as an important cause of escalating health care costs [40]. The 30-dRR is related to the SAE rate in that both measures characterize unanticipated harms to the patient, unplanned expenses to the medical system, and/or unperceived problems in either the patient or the medical system. Yet the 30-dRR should not be construed as a measure of failure, because hospital readmission can represent a successful rescue of the patient from dire consequences of surgery [2]. Rehospitalization may thus reflect good medical judgement rather than bad medical care [2].

The leading causes of SAEs and 30-dRR were surgical complications requiring operative treatment, VTE, and deep infection (Tables 4 and 5). Return to OR constitutes a generalized and composite cause of SAEs and 30-dRR, which cannot be split further to its constituent elements due to lack of available data. With the implementation of strict protocols regarding VTE prevention [41], symptomatic VTE following TKA has reportedly been reduced to 0.3% [42]. Even so, VTE remains among the primary causes of 30-dRR and SAE following TKA. Deep infection is also a problem, despite new protocols that have been developed for TKA, including use of antibiotics [43, 44], preoperative nasal screening for drug-resistant bacteria and subsequent decolonization procedures [45, 46], and strategies to optimize air quality in operating rooms [47, 48].

Readmission following orthopedic surgery was linked to surgical site infection in 25.4% of cases in one study [49]. Reasons for hospital readmission in a sample of 4057 Medicare patients who had TKA were largely medical, rather than surgical [31]. The reported overall 30-dRR was 5.6%, and the 10 most common reasons for readmission were congestive heart failure (20.4%), chronic ischemic heart disease (13.9%), cardiac dysrhythmias (12.5%), pneumonia (10.8%), osteoarthrosis (9.4%), general symptoms (7.4%), acute myocardial infarction (7.0%), care involving other rehabilitation procedures (6.3%), diabetes mellitus (6.3%), and disorders of fluid, electrolyte, and acid-base balance (5.9%). Thus, the top 10 causes of readmission listed did not include VTE [31], which were what we found to be important (Table 2).

We have documented a clear decline of both 30-dRR and SAEs over the study period (Table 3). Many factors have potentially contributed to this result, such as implementation of stricter protocols for VTE prevention, patient decolonization procedures, air quality optimizing strategies in operating theaters, preoperative cardiac clearance, tighter diabetic control, and weight loss programs.

### Limitations and strengths

The results of our primary analysis are characterized by a high degree of statistical heterogeneity, which indicates that patient populations in the pooled studies were not identical. Several included studies were type 2 prognostic factor studies [50] based on large registries. Registries are retrospective, so the populations within them are expected to differ in terms of factors such as baseline and demographic characteristics, comorbidities, interventional protocols, follow-up strategies, and so on. Due to this diversity, outcomes differed among the constituent studies to a greater degree than expected by chance.

Meta-analyses based on retrospective observational studies are prone to bias because they bring together material from many sources, with potential differences in demographic characteristics of included cohorts, inconsistent definitions of outcomes of interest, different surgical and follow-up protocols, and so on. We tried to minimize heterogeneity as an issue by having a focused review question, using strict eligibility criteria, and having clear definitions of outcomes of interest. Because there was significant statistical heterogeneity, we used a random effects model, but the combined estimates of effect size should be interpreted with caution. It is more prudent to consider the 95% confidence intervals rather than the point estimate of each outcome of interest. Only when the pooled estimate of an effect size is derived from high-quality randomized clinical trials, using similar treatment protocols, can an effect size estimate be defined with accuracy. However, in the present report, we synthesize results from a huge group of patients in a statistically valid way, using all existing material from large multicenter series and registries. We believe our conclusions are useful in describing current trends related to short-term outcomes of TKA.

It is possible that 30-dRR may be a more consistent metric than the SAE rate. Comparisons of SAE may be particularly sensitive to the type of database used (administrative claims versus clinical registry), the time period of the comparison, the definitions of variables of interest, and the specific population captured [51]. We therefore explored potential sources of heterogeneity by subgroup analysis. Heterogeneity was markedly reduced in some subgroup comparisons; for example, heterogeneity was 45% in comparing 30-dRR of pTKA to rTKA, but heterogeneity was 0% when pTKA was compared with unicompartmental TKA. Because data pooling within subgroups could be done without significant heterogeneity, this implies that the high initial level of heterogeneity may be a function of several diverse subgroups within the included material. Heterogeneity is often statistically significant in our results because sample sizes are large enough that even small differences become significant.

Several additional limitations of this research are noteworthy: 30-dRR may underestimate actual negative outcomes because procedure-related hospital readmissions—especially for periprosthetic joint infection—could occur with a delay longer than 30 days [52]; facilities with a poor record of 30-day readmission may be reluctant to report this fact in the literature, so the published 30-dRR may underestimate the actual 30-dRR; and some potentially important SAEs, such as opioid addiction, are not generally reported and therefore could not be collated here.

A strength of our study is that the risk of bias in component studies was assessed with the QUIPS tool. Our exploration of publication bias indicates that it was unlikely that we failed to consider reports that could alter our results. Consistent with this, the confidence intervals of our findings are narrow, and the sensitivity analysis suggests that our results are robust.

Another strength of our study is that it may help patients to evaluate the risks, benefits, and timing of TKA more objectively. TKA is generally accepted as the definitive treatment for advanced knee OA after patients fail non-operative treatments [53]. This reasoning suggests that there is no point in postponing the inevitable; if TKA benefits most patients, why not offer that benefit as soon as possible? However, there are several important objections to the strategy of early replacement. First, TKA may not be a definitive treatment for all patients; if patients suffer treatment-related SAEs, as documented here, then additional treatment will be required. Second, hardware failure with time can lead to a need for revision surgery, and revision surgery is more prone to complications than is pTKA (Fig. 2). Postponing surgery until no revision is likely to be needed limits costs and makes sense for the patient, provided that effective symptomatic relief can be offered prior to TKA [54]. Third, patients may opt for premature TKA because of unrealistic expectations of positive outcomes, undervaluation of the risk of negative outcomes, and lack of knowledge about competing treatments [55].

## Conclusions

Our results suggest that despite advances in the surgical technique and implant design, TKA procedures are still characterized by a non-negligible complication rate, which is more pronounced in revision surgery. Leading causes of SAEs and readmissions were surgical complications, VTE events, and deep infections. However, there has been an improvement in SAE and readmission rates over the past decade, suggesting that further improvements in outcome may be expected in years to come.

## Supplementary Information

**Additional file 1.** PRISMA 2009 Checklist.

## Data Availability

The datasets analyzed in the current study are available from the corresponding author on reasonable request.

## Abbreviations

TKA: Total knee arthroplasty
SAE: Serious adverse event
30-dRR: Thirty-day readmission rate
pTKA: Primary TKA
rTKA: Revision TKA
OR: Operating room
NSQIP: National Surgical Quality Improvement Program
VA: Veteran Affairs
CMS: Center for Medicare and Medicaid Services
PRISMA: Preferred Reporting Items for Systematic Reviews and Meta-Analyses
QUIPS: Quality in Prognosis Studies
GRADE: Grading of Recommendations Assessment, Development and Evaluation
VTE: Venous thromboembolism
MI: Myocardial infarction
HF: Heart failure
MESH: Medical Subject Headings
